# Cytotoxic Compounds from Brucea mollis

**DOI:** 10.3797/scipharm.1206-02

**Published:** 2012-12-31

**Authors:** Mai Hung Thanh Tung, Ho Viet Đuc, Tran Thu Huong, Nguyen Thanh Duong, Do Thi Phuong, Do Thi Thao, Bui Huu Tai, Young Ho Kim, Tran The Bach, Nguyen Manh Cuong

**Affiliations:** 1 Institute of Natural Products Chemistry, VAST, 18 Hoang Quoc Viet street, Cau Giay, Hanoi, Vietnam.; 2 Institute of Bioscience and Biotechnology, VAST, 18 Hoang Quoc Viet street, Cau Giay, Hanoi, Vietnam.; 3 College of Pharmacy, Chungnam National University, Daejeon 305-764, South Korea.; 4 Institute of Ecology and Biological Resources, 18 Hoang Quoc Viet street, Cau Giay, Hanoi, Vietnam.

**Keywords:** *Brucea mollis*, Cytotoxicity, Quassinoids, Alkaloids, Triterpenoids

## Abstract

Ten compounds, including soulameanone (**1**), isobruceine B (**2**), 9-methoxy-canthin-6-one (**3**), bruceolline F (**4**), niloticine (**5**), octatriacontan-1-ol (**6**), bombiprenone (**7**), α-tocopherol (**8**), inosine (**9**), and apigenin 7-*O*-β-D-glucopyranoside (**10**), were isolated from the leaves, stems, and roots of *Brucea mollis* Wall. ex Kurz. Their structures were determined using one-and two-dimensional NMR spectroscopy and mass spectrometry. All compounds were evaluated for their cytotoxic activity against KB (human carcinoma of the mouth), LU-1 (human lung adenocarcinoma), LNCaP (human prostate adeno-carcinoma), and HL-60 (human promyelocytic leukemia) cancer cell lines. Compound **2** showed significant cytotoxic activity against KB, LU-1, LNCaP, and HL-60 cancer cells with IC_50_ values of 0.39, 0.40, 0.34, and 0.23 μg/mL, respectively. In addition, compounds 3 and 5 showed significant cytotoxic activity against KB, LU-1, LNCaP, and HL-60 cancer cells with IC_50_ values around 1–4 μg/mL. Compounds 9-methoxycanthin-6-one (**3**) and niloticine (**5**) have been discovered for the first time from the *Brucea* genus.

## Introduction

*Brucea* is a genus belonging to the family Simaroubaceae. It contains about six species mainly distributed in the tropical Eastern Hemisphere and found in Vietnam, China, northern Australia, and other Asian countries [1].

There are six *Brucea* species that have been chemically and biologically characterized: *Brucea javanica*, *Brucea mollis* var*. tonkinensis, Brucea mollis*, *Brucea sumatrana*, *Brucea antidysenterica*, and *Brucea amarissima*. Many compounds have been isolated from this genus, including quassinoids, alkaloids, triterpenoids, and flavonoids. Among these, quassinoids, a chemical class isolated only from the Simaroubaceous species, are the dominant constituents [2]. According to Ho, 2000, the *Brucea* genus in Vietnam consists of three species: *B. javanica*, *B. mollis*, and *B. tonkinensis*[3]. Up until now, there were some reports on the chemistry and bioactivity of *Brucea mollis* in Japan [4] and China [5]. However, the plant *Brucea mollis* collected in Vietnam has not been studied so far.

The results of the present study demonstrated that the methanol extract and n-hexane fraction from *B. mollis* leaves showed strong cytotoxic activity against three cancer cell lines: LU-1 (human lung adenocarcinoma**)**, Hep G2 (liver hepatocellular carcinoma), and MCF-7 (human breast adenocarcinoma) [6].

In this paper, we report the bioactivity-guided isolation of ten compounds, i.e., soulameanone (**1**), isobruceine B (**2**), 9-methoxycanthin-6-one (**3**), bruceolline F (**4**), niloticine (**5**), 1-octatriacontan-1-ol (**6**), bombiprenone (**7**), α-tocopherol (**8**), inosine (**9**), and apigenin 7-*O*-β-D-glucopyranoside (**10**), from the leaves, stems, and roots of *B. mollis*. In addition, we examined the cytotoxic activity of these compounds against KB (carcinoma of the mouth), LU-1 (lung adenocarcinoma), LNCaP (human prostate adenocarcinoma), and HL-60 (promyelocytic leukemia) human cancer cell lines.

## Results and Discussion

Compound **1** was obtained as a white powder. Its molecular formula was determined to be C_20_H_28_O_8_ on the basis of HR-ESI-MS (C_20_H_29_O_8_, *m/z* 397.3403 [M+H]^+^). The ^1^H NMR spectrum of **1** showed resonances ascribable to one olefinic methyl (δ 1.93, H-18), one methyl (δ 0.92, H-21), two tertiary methyls (δ 1.05, H-19; 1.36, H-20), one olefinic proton (δ 5.97, H-3), one methylene (δ 2.08, H-6), and five methines bearing oxygen at δ 3.5–5.0.

The ^13^C and DEPT NMR spectra of **1** revealed 20 carbon signals, including four methyls, one methylene, seven methines, and eight non-hydrogenated carbons. The ^13^C-NMR, HSQC, and HMBC spectra showed that **1** was a quassinoid with a picrasane skeleton. As compared with data reported by Polonsky et al., 1980, **1** was determined to be soulameanone, a quassinoid isolated from *Soulamea muelleri*[7].

Compound **2** was obtained as a yellow powder. The ^13^C-NMR spectra of **2** were similar to those of soulameanone, with additional signals due to the methyleneoxy bridge between the C-8 and C-13 carbons and two substituted groups at the C-13 and C-15 carbons.

The ^13^C and DEPT-NMR (90 and 135) spectra of **2** showed 22 carbon signals, including four methyls, two methylenes, nine methines, and seven non-hydrogenated carbons. The ^1^H-, ^13^C-NMR, DEPT, HSQC, and HMBC spectra of **2** showed one α,β-unsaturated ketone group at δ 196.9 (C-2), 124.3 (C-3), 162.9 (C-4), one ketone group (δ 166.8, C-16), one carbomethoxy group (δ 172.6, 53.0), and one acetyl group (δ 169.0; 20.3).

Compound **2** was a C-20 pentacyclic quassinoid with two side chains at the C-13 and C-15 carbons, as indicated in the HMBC spectra. The mass spectrometric (ESI-MS) data for **2** showed a molecular ion peak at *m/z* 480.7 [M+H]^+^. Based on these data and a comparison with data reported by Fukamiya, 1988 [8], cpd. **2** was determined to be isobruceine B.

Compound **3** was obtained as a yellow powder from the CH_2_Cl_2_ fraction of the stems and roots of *B. mollis.* The molecular formula of **3** was determined to be C_15_H_10_N_2_O_2_ on the basis of HR-ESI-MS (C_15_H_11_N_2_O_2_, *m/z* 251.0820 [M+H]^+^). The ^1^H NMR spectrum of **3** showed one *ortho*-coupled signal at δ 6.93 (d, *J* = 9.5 Hz, H-5), 7.98 (d, *J* = 9.5 Hz, H-4), two vicinal protons at 7.80 (d, *J* = 5.0 Hz, H-1), 8.74 (d, *J* = 5.0 Hz, H-2), three aromatic protons of a 1,3,4-substituted benzene ring at δ 8.16 (d, *J* = 2.0 Hz, H-8), 7.04 (dd, *J* = 8.5, 2.0 Hz, H-10), 7.90 (d, *J* = 8.5 Hz, H-11), and one methoxyl signal at δ 3.98 (s, H-17), corresponding to the carbon at δ 56.0 (C-17). In addition, the HMBC correlation of H_3_-17 methoxyl protons to aromatic carbon C-9 suggested that the position of the methoxyl group was C-9. Based on the HMBC, COSY spectra of **3,** and comparisons with previously reported data [9], compound **3** was determined to be 9-methoxycanthin-6-one.

The ^1^H, ^13^C NMR of **4** showed an indole glycoside structure indicated by the presence of a 3-substituted indole moiety. It was characterized by five aromatic protons at δ 7.54 (d, *J* = 7.5 Hz, H-4), 7.50 (d, *J* = 8.0 Hz, H-7), 7.12 (t, *J* = 7.5 Hz, H-6), 7.03 (t, *J* = 7.5 Hz, H-5), and a singlet proton at δ 7.28 (*s*, H-2). The presence of a β-O-D-glucopyranoside group in **4** was characterized by six carbon signals at δ 84,5 (C-1″), 79,3 (C-5″), 77,5 (C-3″), 71,9 (C-2″), 70,0 (C-4″), 61,0 (C-6″), and an anomeric proton at δ 5.37 (d, *J* = 9.5 Hz, H-1″). Based on ^13^C, ^1^H NMR, HMBC, COSY, and NOESY analysis and comparisons with data reported in the literature [10], compound **4** was determined to be bruceolline F.

Compound **5** was obtained as a yellow powder from the CH_2_Cl_2_ fraction of the stem and roots of *Brucea mollis.* The molecular formula of **5** was determined to be C_30_H_48_O_3_ on the basis of HR-ESI-MS (C_30_H_49_O_3_*m/z*: 457.3682 [M+H]^+^). The ^13^C and DEPT NMR spectra of **5** revealed 30 carbon signals, including eight methyls, eight methylenes, seven methines, six non-hydrogenated carbons, and one carbonyl group. Compound **5** had three carbons bearing oxygen at δ 69.3 (t, C-23), 68.5 (d, C-24), 60.3 (s, C-25), and only two olefinic carbons at δ 118.0 (d, C-7) and 145.7 (s, C-8). Artane triterpene **5** showed spectral patterns indicating the presence of an alkyl 24,25-epoxy side chain at C-17 carbon. Compound **5** was identified as niloticine by comparing its 1D, 2D-NMR spectral data with those in the literature [11].

Some other compounds were also isolated from the leaves, stem, and roots of *B. mollis*, including 1-octatriacontan-1-ol (**6**), bombiprenone (**7**), α-tocopherol (**8**), one nucleoside, inosine (**9**), and one flavonoid, apigenin 7-*O*-β-D-glucopyranoside **(10**). Structures of all of the isolated compounds were determined on the basis of ^1^H, ^13^C NMR, and ESI-MS spectra, and were compared with references [12–14].

**Fig. 2:**
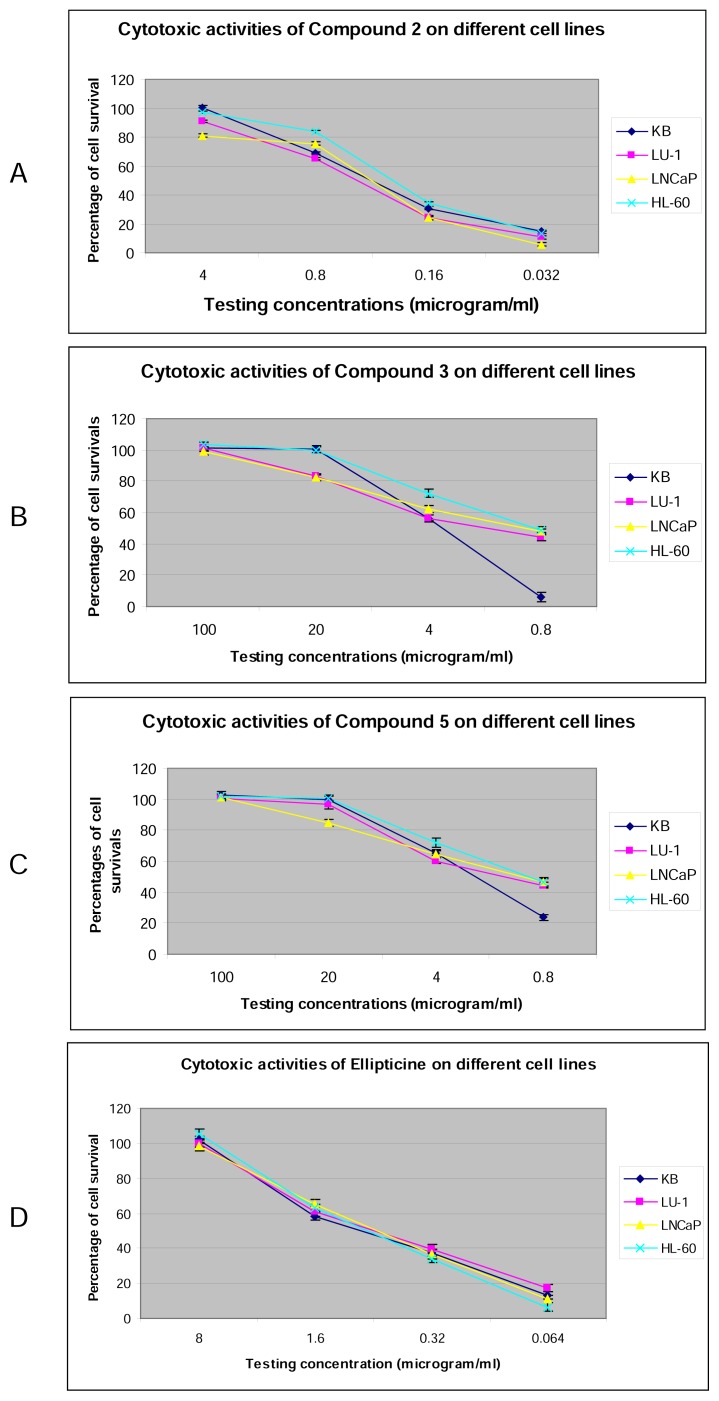
A: cytotoxic activities of compound **2**; B: cytotoxic activities of compound **3**; C: cytotoxic activities of compound **5**; D: cytotoxic activities of ellipticine.

The inhibitory activity of the isolated compounds was assessed using a cytotoxic assay against four cancer cell lines: KB (human carcinoma of the mouth), LU-1 (human lung adenocarcinoma), LNCaP (human prostate adenocarcinoma), and HL-60 (human promyelocytic leukemia). Those cell lines were selected based on the reason that KB cells were usually the classical cells used for all cytotoxic assays. Also, the others were employed in order to discover new activities of the tested compounds. The results showed that isobruceine B (**2**), 9-methoxycanthin-6-one (**3**), and niloticine (**5**) possessed strong inhibitory activities toward the tested cancer cell lines, with low IC_50_ values ranging from 0.23–0.4 μg/mL, 0.91–3.73 μg/mL, and 1.00–2.22 μg/mL, respectively (see Table 1, Fig. 2). In particular, the cancerous cellular growth inhibitory activity of isobruceine B (**2**) was stronger than that of ellipticine, the positive control (IC_50_ values ranging from 0.66–0.89 μg/mL). Isobruceine B was isolated from the wood *of Brucea antidysenterica*[15] with no biological activity reported, and from the stems of the Simaroubaceae *Picrolemma sprucei* plant, which was used in the Amazon regions of Peru, Brazil, and French Guiana as antimalarials [16]. Isobruceine B exhibited *in vitro* cytotoxic activities against SF-295, HCT-8, and HL-60 human tumor cells lines (IC_50_ = 5–27 μg/L), and against the malarial multidrug-resistant *Plasmodium falciparum* K1 strain (IC_50_ = 1.0–4.0 μg/L) [17].

Compound **2** had structural patterns of an anticancer quassinoid, including a four-ring skeleton with a lactone D-ring, an α,β-unsaturated ketone in the A-ring, two free hydroxyl groups, an oxygen-methylene bridge in the C-ring, and an ester group at either the C-13 or C-15 position [18].

Some canthin-6-one alkaloids, commonly found in *Eurycoma* and *Picrasma* plants (Simaroubaceae family), showed *in vitro* inhibition of several cancer cell lines, including A-388, A549, MCF-7, and Bel-7402 [19, 20].

It is interesting to note that three cytotoxic compounds of different skeletons, i.e., quassinoid isobruceine B, alkaloid 9-methoxycanthin-6-one, and triterpene niloticine, were found in the *Brucea mollis* plant. Of those, 9-methoxycanthin-6-one (**3**) and niloticine (**5**) have been discovered for the first time from *Brucea* genus.

## Experimental

### General

The 1D and 2D-NMR experiments were recorded on a Bruker Avance 500 (500 MHz for ^1^H-NMR and 125 MHz for ^13^C-NMR), using DMSO-*d_6_* and CDCl_3_ as solvents. ESI-MS was recorded on the Agilent 6310 Ion Trap, and HR-ESI-MS was recorded on the Agilent 6510 Q-TOF LC/MS. HPLC analysis was carried out on the Waters HPLC system, with the Waters 600 controller, Waters 600 pump, and Waters 996 photodiode array detector. Silica gel (70–230, 230–400 mesh, Merck), and YMC RP-18 resins (30–50 μm, Fuji Silysia Chemical Ltd.) were used as absorbents in the column chromatography. Thin-layer chromatography (TLC) plates (Silica gel 60 F_254_ and RP-18 F_254_, 0.25 μm, Merck) were purchased from Merck KGaA (Darmstadt, Germany). Spots were detected under UV radiation (254 and 365 nm) and by spraying the plates with 10% H_2_SO_4_ followed by heating with a heat gun.

### Plant material

*Brucea mollis* (Wall. ex Kurz) was collected in March 2009 in the Hoa Binh province, Vietnam. The scientific name was identified by Dr. Tran The Bach, Institute of Ecology and Biological Resources, Vietnam Academy of Science and Technology. A voucher specimen (VK 2211(HN)) has been deposited in the herbarium of Institute of Ecology and Biological Resources.

### Extraction and isolation

Dried powder from the leaves (2.6 kg), stems, and roots (8.6 kg) of *B. mollis* were extracted at room temperature with MeOH (three times × 72 h). The solvent was removed under reduced pressure to obtain a residue (191 g) of leaves and a residue (300 g) of the stem and roots. These residues were suspended in water and partitioned with *n*-hexane, CH_2_Cl_2_, and EtOAc, successively.

A mixture of n-hexane and CH_2_Cl_2_ fractions from the leaves (100 g) was subjected to column chromatography (φ = 15 cm, height of silica gel 4 cm) over silica gel and eluted with CH_2_Cl_2_–MeOH (100:0, 40:1, 20:1, 10:1, 5:1, 2.5:1, 1:1, 0:100) to obtain eight fractions (1L each fraction). Fraction 3 (27 g) was separated by column chromatography over silica gel (φ = 10 cm, height of silica gel 20 cm) and eluted with CH_2_Cl_2_–MeOH (gradient, 1–100%, flow rate 5 ml/min) to obtain 11 sub-fractions (Fr.1–Fr.11, 500 ml each fraction). Sub-fraction 6 (9 g) produced a precipitate of crude crystals, which was separated by filtering and washing with acetone to yield compound **6** (22 mg). Sub-fraction 2 (5 g) was separated by column chromatography (φ = 2.5 cm, height of silica gel 70 cm) and eluted with n-hexane-EtOAc (40:1, flow rate 2 ml/min) to yield compound **7** (70 mg) and compound **8** (75 mg).

After evaporation of the solvent, the EtOAc fraction from the leaves (8 g) was subjected to column chromatography over silica gel (φ = 3 cm, height of silica gel 60 cm) and eluted with acetone–CHCl_3_–MeOH (3:1:0.1) to obtain eight fractions (Fr.1–Fr.8). Fraction 2 (1 g) was separated over a YMC RP-18 column (φ = 2 cm, height of silica gel 60 cm) with MeOH-water (1:4, flow rate 0.5 ml/min) to yield compound **1** (60 mg). Fraction 4 (1.5 g) produced a precipitate of crude yellow crystals, which was separated by filtering and washing with MeOH to yield compound **10** (280 mg).

The CH_2_Cl_2_ fraction of the stems and roots (85 g) was subjected to column chromatography over silica gel (φ = 15 cm, height of silica gel 4 cm) and eluted with CH_2_Cl_2_–MeOH (100:0, 40:1, 20:1, 10:1, 5:1, 2.5:1, 1:1, 0:100), to obtain eight fractions (Fr.4A–4H, 1L each fraction). Fractions 4A and 4B were combined (31.5 g) and separated over silica gel (φ = 10 cm, height of silica gel 20 cm) with n-hexane-acetone (3:1, 2:1, 1:1, flow rate 5 ml/min) to obtain nine fractions (Fr.5A–5I, 500 ml each fraction). Fraction 5I (2 g) was separated over silica gel (φ = 2 cm, height of silica gel 60 cm) with CH_2_Cl_2_-EtOAc (6:1, 5:1, 4:1, 1:1, flow rate 2.5 ml/min) to yield compound **2** (70 mg).

Fraction 5C (3.6 g) was separated over silica gel (φ = 2 cm, height of silica gel 60 cm) with *n*-hexane–EtOAc 6:1 to obtain seven fractions (Fr.6A–6G). Fraction 6E (120 mg) was separated over silica gel (φ = 1.5 cm, height of silica gel 60 cm) with n-hexane-EtOAc (6:1, flow rate 1.5 ml/min) to yield compound **3** (50 mg). Fraction 6G (250 mg) was separated with a YMC RP-18 column (φ = 2 cm X 60 cm) and eluted with MeOH-water (1:1.5, 2:1, 4:1, flow rate 0.5 ml/min) to yield compound **5** (100 mg).

The water fractions of the stems and roots (150 g) were subjected to the Dianion HP-20 column (φ = 10 cm, height of materials 30 cm) and eluted with stepwise increases of MeOH in water (25, 50, 75, and 100%, 1L each fraction). The fraction eluted by 25% MeOH (9 g) was separated over silica gel (φ = 3 cm, height of silica gel 60 cm) with CH_2_Cl_2_-MeOH-water (4:1:0.1, flow rate 2.5 ml/min) to obtain 12 fractions. Fraction 11 (500 mg) was separated by column chromatography over silica gel (φ = 2 cm, height of silica gel 60 cm) and eluted with acetone-diclometan-water (3.5:1:0.1, flow rate 2 ml/min) to yield compound **9** (26 mg).

The fraction eluted by 50% MeOH (18 g) was separated over silica gel (φ = 2 cm, height of silica gel 60 cm) with CH_2_Cl_2_–MeOH-water (4:1:0.1, flow rate 2 ml/min) to obtain nine fractions (Fr.7A–7I). Fraction 7D (2.6 g) produced a precipitate of crude crystals, which was separated by filtering and washing with MeOH to yield compound **4** (200 mg). The purity of isolated compounds was more than 95% by HPLC analysis.

### Soulameanone ((1β,11β,12β,15β)-1,11,12,13,15-Pentahydroxypicras-3-ene-2,16-dione, 1)

White powder of mp 263–265°C, C_20_H_28_O_8_. ^1^H-NMR (500 MHz, DMSO-*d_6_*): δ 6.29 (bs, 15-OH), 5.97 (s, H-3), 5.69 (bs, 13-OH), 4.86 (bs, H-7), 4.78 (bs, 12-OH), 4.53 (bs, 1-OH), 4.27 (dd, *J* = 6.5, 2.0 Hz, H-15), 4.13 (s, H-1), 4.07 (bs, H-11), 4.07 (bs, 11-OH), 3.47 (dd, *J* = 6.5, 2.5 Hz, H-12), 2.88 (t, *J* = 6.5 Hz, H-5), 2.08 (m, H-6), 1.95 (s, H-9), 1.95 (s, H-14), 1.93 (s, H-18), 1.36 (s, H-20), 1.05 (s, H-19), 0.92 (s, H-21). ^13^C-NMR **(**DMSO-*d_6_*, 125 MHz): δ 198.2 (s, C-2), 171.4 (s, C-16), 164.0 (s, C-4), 124.3 (d, C-3), 82.1 (d, C-1), 78.1 (d, C-7), 78.0 (d, C-12), 74.8 (d, C-11), 71.8 (s, C-13), 66.7 (d, C-15), 60.5 (d, C-14), 47.1 (s, C-10), 44.8 (d, C-9), 42.6 (d, C-5), 37.8 (s, C-8), 28.8 (q, C-20), 24.4 (t, C-6), 21.9 (q, C-187), 17.8 (q, C-21), 12.9 (q, C-19). HRESIMS: calcd for C_20_H_29_O_8_: 397.4315. found: 397.1862.

### Isobruceine B (Methyl (1β,11β,12α,15β)-15-(acetyloxy)-1,11,12-trihydroxy-2,16-dioxo-13,20-epoxypicras-3-en-21-oate, 2)

Yellow powder of mp 243–246°C, C_23_H_28_O_11_. ^1^H-NMR (500 MHz, CDCl_3_): δ 6.34 (bs, H-15), 6.11 (s, H-3), 4.81 (d, *J* = 7.5 Hz, H-20β), 4.75 (d, *J* = 3.0 Hz, H-11), 4.74 (d, *J* = 3.0 Hz, H-7), 4.28 (d, *J* = 1.0 Hz, H-12), 4.17 (s, H-1), 2.92 (d, *J* = 12.5 Hz, H-5), 3.83 (s, H-1′), 3.75 (dd, *J* = 8.0, 1.5 Hz, H-20α), 3.04 (d, *J* = 12.5 Hz, H-14), 2.41 (dd, *J* = 14.5, 3.0 Hz, H-6β), 2.33 (d, *J* = 3.5 Hz, H-9), 2.11 (s, H-2″), 1.95 (s, H-18), 1.86 (ddd, *J* = 13.0, 2.5, 2.5 Hz, H-6α), 1.18 (s, H-19). ^13^C-NMR **(**CDCl_3_, 125 MHz): δ 196.9 (s, C-2), 172.6 (s, C-21), 169.0 (s, C-1″), 166.8 (s, C-16), 162.9 (s, C-4), 124.3 (d, C-3), 83.0 (d, C-7), 81.1 (d, C-1), 80.5 (s, C-13), 75.8 (d, C-12), 73.3 (t, C-20), 72.4 (d, C-11), 67.0 (d, C-15), 53.0 (q, C-1′, OCH_3_), 51.5 (d, C-14), 47.5 (s, C-10), 45.6 (s, C-8), 43.5 (d, C-5), 42.8 (d, C-9), 28.5 (t, C-6), 22.5 (q, C-18), 20.3 (q, C-2″), 11.5 (q, C-19). ESIMS (*m/z*, %): 480.7 ([M+ H]^+^, 100).

### 9-Methoxycanthin-6-one (9-Methoxy-6H-indolo[3,2,1-de][1,5]naphthyridin-6-one, 3)

Yellow powder of mp 178–180°C, C_15_H_10_N_2_O_2_. ^1^H-NMR (500 MHz, CDCl_3_): δ 8.74 (d, *J* = 5.0 Hz, H-2), 8.16 (d, *J* = 2.0 Hz, H-8), 7.98 (d, *J* = 9.5 Hz, H-4), 7.90 (d, *J* = 8.5 Hz, H-11), 7.80 (d, *J* = 5.0 Hz, H-1), 7.04 (dd, *J* = 8.5, 2.0 Hz, H-10), 6.93 (d, *J* = 9.5 Hz, H-5), 3.97 (s, H-17). ^13^C-NMR (125 MHz, CDCl_3_): δ 162.5 (s, C-9), 159.7 (s, C-6), 146.0 (d, C-2), 141.2 (s, C-13), 139.9 (d, C-4), 135.7 (s, C-16), 132.3 (s, C-15), 130.4 (s, C-14), 128.5 (d, C-5), 123.3 (d, C-11), 117.2 (s, C-12), 115.5 (d, C-1), 114.2 (d, C-10), 101.3 (d, C-8), 56.0 (q, C-17). HRESIMS: calcd for C_15_H_11_N_2_O_2_: 251.0742. found: 251,0820.

### Bruceolline F (1-(1-β-D-glucopyranosyl-1H-indol-3-yl)-3-methylbutane-2,3-diol, 4)

Yellow powder of mp 209–211°C, C_19_H_27_NO_7_. ^1^H-NMR (500 MHz, DMSO-*d_6_*): δ 7.54 (d, *J* = 7.5 Hz, H-4), 7.50 (d, *J* = 8,0 Hz, H-7), 7.28 (s, H-2), 7.12 (t, *J* =7.5 Hz, H-6), 7.03 (t, *J* = 7.5 Hz, H-5), 5.37 (d, *J* = 9.5 Hz, H-1″), 5.18 (d, *J* = 5.0 Hz, 3″-OH), 5.10 (d, *J* = 5.5 Hz, 2″-OH), 5.10 (d, *J* = 5.5 Hz, 4″-OH), 4.56 (t, *J* = 5.5 Hz, 6″-OH), 4.34 (d, *J* = 5.5 Hz, 2′-OH), 4.29 (s, 3′-OH), 3.73 (dt, *J* = 8.5, 5.5 Hz, H-2″), 3.70 (ddd, *J* = 13.5, 9,0, 3.5 Hz, H-6″a), 3.46 (m, H-5″), 3.45 (m, H-6″b), 3.44 (d, *J* =10.5 Hz, H-2′), 3.42 (dt, *J* = 9.0, 5.0 Hz, H-3″), 3.26 (dt, *J* = 9.0, 5.5Hz, H-4″), 3.05 (d, *J* =15.0 Hz, H-1′a), 2.46 (dd, *J* = 14.5, 10.5 Hz, H-1′b), 1.17 (s, H-5′), 1.14 (s, H-4′). ^13^C-NMR (125 MHz, DMSO-d_6_): δ 136.5 (s, C-8), 128.6 (s, C-9), 123.7 (d, C-2), 121.0 (d, C-6), 118.8 (d, C-5), 118.8 (d, C-4), 113.8 (s, C-3), 110.5 (d, C-7), 84.5 (d, C-1″), 79.3 (d, C-5″), 77.8 (d, C-2′), 77.5 (d, C-3″), 71.9 (d, C-2″), 71.8 (s, C-3′), 70.0 (d, C-4″), 61.0 (t, C-6″), 27.0 (t, C-1′), 26.9 (q, C-5′), 24.2 (q, C-4′). ESIMS (*m/z*, %): 418.0 ([M+2H_2_O+H]^+^, 100).

### Niloticin (13α,14β,20S,23R,24S)-23-Hydroxy-24,25-epoxylanost-7-en-3-one, 5)

Yellow powder of mp 147–149°C, C_30_H_48_O_3_. ^1^H-NMR (500 MHz, CDCl_3_) δ 5.32 (m, H-7), 3.57 (m, H-23), 2.76 (dt, *J* = 14.5, 5.5 Hz, H-1a), 2.66 (d, *J* = 8.5 Hz, H-24), 2.23 (t, *J* = 3.5 Hz, H-1b), 2.27 (t, *J* = 3.5 Hz, H-9), 2.10 (t, *J* = 3.5 Hz, H-6), 2.05 (m, H-16a), 2.02 (m, H-2a), 1.82 (m, H-12a), 1.72 (d, *J* = 8.5 Hz, H-5), 1.66 (m, H-22a), 1.65 (m, H-20), 1.64 (m, H-11), 1.58 (m, H-17), 1.50 (m, H-15), 1.49 (m, H-2b), 1.44 (m, H-22b), 1.42 (m, H-12b), 1.33 (s, H-26), 1.32 (s, H-27), 1.23 (m, H-16b), 1.12 (s, H-29), 1.05 (s, H-28), 1.03 (s, H-30), 1.01 (s, H-19), 0.96 (d, *J* = 6.0 Hz, H-21), 0.82 (s, H-18). ^13^C-NMR (125 MHz, CDCl_3_): δ 216.9 (s, C-3), 145.7 (s, C-8), 118.0 (d, C-7), 69.3 (t, C-23), 68.5 (d, C-24), 60.3 (s, C-25), 53.3 (d, C-17), 52.4 (d, C-5), 51.2 (s, C-14), 48.5 (d, C-9), 47.9 (s, C-4), 43.6 (s, C-13), 40.7 (t, C-22), 38.6 (t, C-2), 35.0 (s, C-10), 34.9 (t, C-1), 34.0 (t, C-15), 33.6 (t, C-12), 33.6 (d, C-20), 28.8 (t, C-16), 27.4 (q, C-30), 24.9 (q, C-27), 24.6 (q, C-28), 24.4 (t, C-6), 21.8 (q, C-18), 21.6 (q, C-29), 19.9 (q, C-2), 19.8 (q, C-26), 18.3 (t, C-11), 12.8 (q, C-19). HRESIMS: calcd for C_30_H_49_O_3_: 457.3603. found: 457.3682.

### Octatriacontan-1-ol (6)

White powder, C_38_H_78_O. ^1^H-NMR (400 MHz, CDCl_3_): δ 3.49 (2H, -CH_2_-O), 1.45 (2H, m, CH_2_), 1.16 (70H, 35CH_2_), 0.78 (3H, m, CH_3_). ^13^C-NMR (100 MHz, CDCl_3_): δ 62.3 (t, -CH_2_-O-), 22.4–32.3 (t, 36CH_2_), 13.7 (q, CH_3_). ESIMS (*m/z*, %): 550.6 ([M+H]^+^, 100)

### Bombiprenone (5E,9E,13E,17E,21E,25E,29E)-6,10,14,18,22,26,30,34-Octamethylpentatriaconta-5,9,13,17,21,25,29,33-octaen-2-one, 7)

Yellow powder, C_43_H_70_O. ^1^H-NMR (500 MHz, CDCl_3_): δ 5.10–5.13 (7H, H-9, H-13, H-17, H-21, H-25, H-29, H-33), 5.08 (1H, H-5), 2.45 (2H, t, *J* = 7.5 Hz, H-3), 2.27 (2H, t, *J* = 7.5 Hz, H-4), 2.13 (3H, s, H-1), 2.05–2.09 (14H, t, *J* = 7.5 Hz, H-8, H-12, H-16, H-20, H-24, H-28, H-32), 1.97–2.00 (14H, t, *J* = 7.5 Hz, H-7, H-11, H-15, H-19, H-23, H- 27, H-31), 1.57–1.60 (21H, s, H-34÷40), 1,68 (3H, s, H-42), 1,62 (3H, s, H-43). ^13^C-NMR (125 MHz, CDCl_3_): δ 208.8 (s, C-2), 134.9–136.5 (s, C-6, C-10, C-14, C-18, C-22, C-26, C-30), 131.2 (s, C-41), 124.1–124.4 (d, C-9, C-13, C-17, C-21, C-25, C-29, C-33), 122.5 (d, C-5), 43.8 (t, C-3), 39.7 (t, C-7, C-11, C-15, C-19, C-23, C-27, C-31), 29.9 (q, C-1), 26.6–26.8 (t, C-8, C-12, C-16, C-20, C-24, C-28, C-32), 25.7 (q, C-42), 22.5 (t, C-4), 17.7 (q, C-43), 16.0 (q, C-34÷40). ESIMS (*m/z*, %): 603.0 ([M+H]^+^, 100).

### α-Tocopherol (2,5,7,8-tetramethyl-2-[(4R,8R)-4,8,12-trimethyltridecyl]-3,4-dihydro-2H-chromen-6-ol, 8)

Red powder, C_29_H_50_O_2_. ^1^H-NMR (500 MHz, CDCl_3_): δ 2.60 (t), 2.16 (s), 2.11 (s), 1.72–1.84 (m), 1.47–1.57 (m), 1.21–1.39 (m), 1.22 (3H, s), 1.05–1.16 (m), 0.84 (d, *J* = 4.5 Hz), 0.86 (d, *J* = 5.0 Hz), 0.87. ^13^C-NMR (125 MHz, CDCl_3_): δ 145.6 (s, C-8a), 144.5 (s, C-6), 122.6 (s, C-8), 121.0 (s, C-7), 118.5 (s, C-5), 117.4 (s, C-4a), 74.5 (s, C-2), 39.8 (t, C-1′), 39.4 (t, C-11′), 37.3–37.5 (t, C-3′, C-5′, C-7′, C-9′), 32.8 (d, C-4′, C-8′), 31.6 (t, C-3), 28.0 (d, C-12′), 24.8 (t, C-10′), 24.5 (t, C-6′), 23.8 (q, C-2a), 22.6 (q, C-12′a), 22.7 (q, C-13′), 21.1 (t, C-2′), 20.8 (t, C-4), 19.8 (q, C-4′a, C-8′a), 12.2 (q, C-7a), 11.8 (q, C-8b), 11.3 (q, C-5a). ESIMS (*m/z*, %): 431.6 ([M+H]^+^, 100).

### Inosine (9-β-D-Ribofuranosyl-9H-purin-6-ol, 9)

White powder, mp. 215°C, C_10_H_12_N_4_O_5_. ^1^H-NMR (500 MHz, CD_3_OD): δ 8.33 (s, H-8), 8.21 (s, H-2), 6.00 (d, *J* = 6.5 Hz, H-1′), 4.76 (t, *J* = 5.8 Hz, H-2′), 4.36 (dd, *J* = 5.0, 2.5 Hz, H-3′), 4.22 (q, *J* = 2.5 Hz, H-4′), 3.91 (dd, *J* = 13.0, 2.5 Hz, H-5′a), 3.79 (dd, *J* = 12.5, 2.5 Hz, H-5′b). ^13^C-NMR (125 MHz, CD_3_OD): δ 157.4 (s, C-6), 153.5 (d, C-2), 149.9 (s, C-4), 142.0 (d, C-8), 120.9 (s, C-5), 91.0 (d, C-1′), 88.0 (d, C-4′), 75.4 (d, C-2′), 72.5 (d, C-3′), 63.3 (t, C-5′). ESIMS (*m/z*, %): 269.5 ([M+H]^+^, 100)

### Apigenin 7-O-β-D-glucopyranoside (5-Hydroxy-2-(4-hydroxyphenyl)-4-oxo-4H-chromen-7-yl β-D-glucopyranoside, 10)

Yellow powder, mp. 238–239°C, C_21_H_20_O_10_. ^1^H-NMR (500 MHz, DMSO-*d_6_*): δ 12.97 (s, 5- OH), 10.37 (s, 4′-OH), 7.95 (d, *J* = 9.0 Hz, H-5′), 6.93 (d, *J* = 8.5 Hz, H-6′), 6.87 (s, H-3), 6.83 (d, *J* = 2.0 Hz, H-6), 6.44 (d, *J* = 2.0 Hz, H-8), 5.06 (d, *J* = 7.5 Hz, H-1″). ^13^C-NMR (125 MHz, DMSO-d_6_): δ 182.0 (s, C-4), 164.3 (s, C-7), 163.0 (s, C-5), 161.1 (s, C-2, C-9), 156.9 (s, C-4′), 128.6 (d, C-2′, C-5′), 121.0 (s, C-1′), 116.0 (d, C-3′, C-6′), 105.3 (s, C-10), 103.1 (d, C-3), 99.9 (d, C-1″), 99.5 (d, C-6), 94.8 (d, C-8), 77.2 (d, C-5″), 76.4 (d, C-3″), 73.1 (d, C-2″), 69.5 (d, C-4″), 60.6 (t, C-6″).

### Cytotoxicity assay

KB (human carcinoma of the mouth), LU-1 (human lung adenocarcinoma), and LNCaP (human prostate adenocarcinoma) cell lines, obtained from ATCC (American Type Culture Collection, Manassas, VA), were maintained as monolayers in DMEM supplemented with 10% fetal bovine serum (FBS; GIBCO, Grant Island, NY), sodium bicarbonate, penicillin G, and streptomycin at 37°C under a humidified atmosphere of 5% CO_2_. HL-60 (human promyelocytic leukemia) cells were grown suspensively in RPMI-1640 medium under the same conditions as the other cell lines. The cytotoxic activity of the tested compounds was measured using the sulforhodamine B (SRB) method [21]. Viable cells were seeded (180 μL) in 96-well microtiter plates at a concentration of 3 × 10^4^ cells/mL and allowed to attach overnight. The test samples were dissolved in 10% DMSO and adjusted to a final working concentration of 100 μg/mL by diluting with the growth medium. Samples were prepared in triplicate. The final DMSO concentration was adjusted to less than 0.1%. The assay duration was three days. One plate without samples served as a 0-day control. Test plates were incubated in a humidified atmosphere of 5% CO_2_ at 37°C for another 72 h, while the 0-day control was incubated for 1 h. After incubation, the medium was removed, except for the HL-60 cell experiments. The remaining cells were fixed using 10% trichloroacetic acid (TCA) for 1 h at 4°C. Plates containing HL-60 cells were fixed using 70% TCA for 2 h at 4°C. The TCA-treated cells were then washed extensively with water and dried in air. Once fixed cell plates dried completely, 50 μL of SRB solution (0.4% in acetic acid) was added to each well at room temperature. After standing for 1 h, the wells were washed 3–4 times with 1% acetic acid and dried in air. Subsequently, the bound dye was solubilized by the addition of 10 mmol of unbuffered Tris base (Sigma, Louis, MO). The absorption was measured at 515 nm with a microplate reader (BioRad, Hercules, CA). Growth, expressed as a percentage of the negative control, was calculated as follows: % growth = [absorbance (test substance) − absorbance (0-day control)] * 100/[absorbance (negative control) − absorbance (0-day control)].

The IC_50_ values (inhibitory concentration at 50%) were determined by plotting concentrations against % growth using nonlinear regression analysis (TableCurve software, San Jose, CA).

## Figures and Tables

**Fig. 1:**
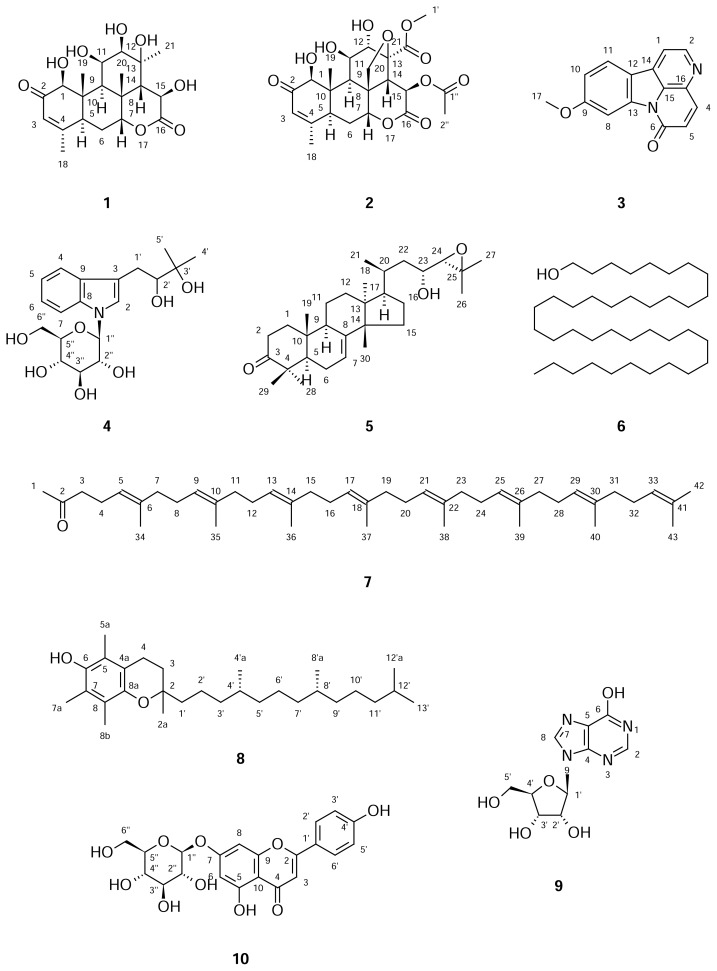
Compounds **1–10**

**Tab. 1:** Cytotoxicity of compound **1–10** against various cancer cell lines

Compound	IC_50_ (μg/ml)			
	
	KB	LU-1	LNCaP	HL-60
**1**	>20	>20	>20	>20
**2**	**0.39 ± 0.02**	**0.40 ± 0.01**	**0.34 ± 0.01**	**0.23 ± 0.03**
**3**	**3.73 ± 0.07**	**1.61 ± 0.06**	**1.01 ± 0.01**	**0.91 ± 0.01**
**4**	> 20	> 20	> 20	> 20
**5**	**2.22 ± 0.03**	**1.22 ± 0.03**	**1.10 ± 0.04**	**1.00 ± 0.02**
**6**	> 20	> 20	> 20	> 20
**7**	> 20	> 20	> 20	> 20
**8**	> 20	> 20	> 20	> 20
**9**	> 20	> 20	> 20	> 20
**10**	> 20	> 20	> 20	> 20
Ellipticine	**0.89 ± 0.02**	**0.72 ± 0.01**	**0.70 ± 0.02**	**0.66 ± 0.02**
